# Turns around periodic spatial boundaries facilitate increasing event segmentation over time

**DOI:** 10.1098/rsos.240835

**Published:** 2024-11-20

**Authors:** Tyler Wayne Ross, Benjamin Slater, Alexander Easton

**Affiliations:** ^1^ Department of Psychology, Durham University, Durham, UK; ^2^ Centre for Learning and Memory Processes, Durham University, Durham, UK; ^3^ Biosciences Institute, Newcastle University, Newcastle upon Tyne, Tyne and Wear, UK

**Keywords:** event segmentation, spatial boundaries, episodic memory, episodic-like memory, pattern separation

## Abstract

Event segmentation is a neurocognitive process bridging perception and episodic memory. To our knowledge, almost all segmentation work is framed towards humans, yet evolutionarily conserved mechanisms in event cognition exist across species. Here, we addressed segmentation in a way that is applicable to humans and non-human animals, inspired by research in rats; specifically, the fragmentation of grid-cell spatial representations following the insertion of boundaries into an environment (forming a corridor maze). Participants indicated when they felt a meaningful unit of activity ended and another began, while watching an agent traverse from a first-person perspective. A virtual corridor maze (experiment 1) and two other mazes were used (experiment 2), with participants viewing/segmenting the same stimuli twice. We found that people segmented more during turns relative to corridors, with elevated segmentation occurring in discrete moments around turns. Interestingly, we also found that boundaries of the corridor maze facilitated an increase in segmentation within and across viewings. These results suggest that segmentation can be driven by recognized repeating activity that can become more meaningful over time, highlighting an important link between event segmentation and pattern separation that is relevant to many species in their formation of episodic-(like) memory.

## 1. Introduction

Despite experiencing a continuous stream of input as we go about our waking daily lives, our memory is fragmented into discrete units via episodic neurocognitive processing [1,2]. Event segmentation occurs at an ‘event boundary’ where one meaningful unit of activity ends and another begins [3,4]. People can have both fine- and coarse-grained event segmentation [4,5] that is reflected in hierarchal cortical activity [6]. Accumulating work has also suggested that the experience of event boundaries is important for subsequent memory formation or lack thereof [7–14]. Thus, what cues event segmentation has become a crucial question in neurocognitive research.

Extensive research based upon text and movie stimuli has highlighted that aspects such as time, space, entity, causation and motivation are key for event representations and segmentation [15]. In text and movie stimuli there is usually a prominent role for narratives, encompassing several of these aspects. While the comprehension and communication of narratives are not only a crucial component of human episodic memory [16,17] but also promote cooperation within human groups, better achieving shared goals [18], the widespread use of narrative-based stimuli has biased event segmentation research towards humans (cf. [19]). This is problematic as many non-human animal species show evidence of episodic memory [20–24], suggesting that there are evolutionarily conserved neurocognitive mechanisms shared across species [21]. Thus, there is a need for more comparable approaches to understand the links between event segmentation and episodic memory.

Transitioning between spatial contexts may cue segmentation of events in both humans and non-human animals [1,25,26]. For example, making goal-directed turns around spatial boundaries in a virtual reality environment facilitated distortions of spatio-temporal cognition [27]. Indeed, the number of turns made when navigating between two real-world landmarks attenuates the mental route compression during navigation memory recall [28]. People have better recollection of images of scenes when tested before a turn relative to mid-route or after the turn [29]. Together, it seems that turns have a prominent role in bounding experiences in spatial memory. Notably, other changes in spatial context such as walking through (or even anticipating walking through) a doorway into a distinct room can also impact episodic memory formation [9,30–32].

Typical experiments in event segmentation have used explicit segmentation tasks [3,4], instructing participants to indicate when they consider one unit of activity ends and another begins. A benefit of these explicit approaches is that one can better examine the extent to which people are spontaneously converging upon similar moments to bound events. This is important as one’s tendency to segment when many other people also segment is predictive of subsequent episodic memory performance [33]. There is, however, mixed evidence for spatial shifts eliciting such event segmentation (e.g. [34–36]). For instance, spatial shifts were only found to be influential when coupled with temporal or action shifts in some movie stimuli, which also had ongoing narratives [34,35]. Hence, we opted to use a segmentation task that approached event segmentation in a more implicit way that could be, in principle, applicable to many species and agents.

**Figure 1.**
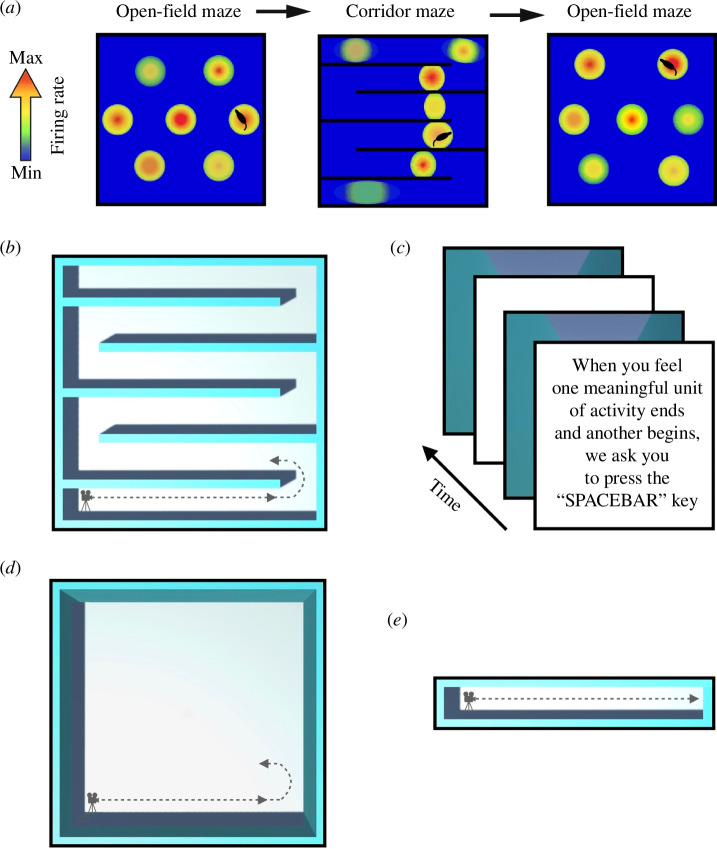
Schematics displaying the inspiration for and the experimental design of the present experiments. (*a*) Simplified schematic based upon [37]. Left depicts a schematic single grid-cell example (not real data), which displays triangular periodic firing fields as animals traverse the open-field. Middle, inserted inner boundaries form corridors (we refer to this as the corridor maze), influencing grid-cell firing by forming discrete spatial representations for corridors, being reset by rats’ turns into proceeding corridors. Right, the typical grid-cell firing pattern returns when rats are placed into the open-field. (*b*) Participants passively viewed an agent traversing a fixed path from a first-person perspective in the corridor maze (birds eye view shown). The dotted line/arrow denotes the example path of the agent. (*c*) Participants’ instructions for experiment 1; they viewed the same stimulus twice with a break and repetition of the instructions in between. (*d*) The open-field maze environment used in experiment 2. (*e*) The elongated corridor used in experiment 2.

Fragmentation of spatial representations in the rodent hippocampal formation can be created by physical boundaries [37]. Specifically, grid-cells display periodic triangular patterned firing fields as rodents traverse open spaces [38], and a grid-like activity also exists in humans during virtual and imagined navigation [39–41]. Yet, Derdikman and colleagues [37] inserted physical boundaries into a maze, creating compartmentalized spaces (a corridor maze), finding that the spatially modulated firing of grid-cells was ‘reset’ as rats turned into corridor arms, thereby forming spatial submaps for each corridor. We sought to use a similar corridor maze design to investigate how people would spontaneously segment when watching an agent traverse a corridor maze (figure 1), building the connection across work in rodents and humans; however, it is important to consider underlying theories as to why animals segment continuous experience.

Event segmentation theory addresses event cognition broadly [7]. Despite event segmentation theory being framed towards humans, it can also explain why segmentation occurs across animals during spatial context changes, in narratives and in other situations. A role for structured knowledge representations of how certain situations generally work, schema [42], is outlined in this theory. Such knowledge can be used as a basis to inform one’s predictions of unfolding experiences in the upcoming future [5,7]. When there is a mismatch (or an accumulation of mismatches) between expectation and reality, a ‘prediction-error’ arises. It is adaptive, then, to update the event model, learning and remembering from that new experience to minimize future prediction-error [5,7,43]. Therefore, event segmentation is likely in these unexpected situations (although event segmentation can also result from perceptual input and can form predictions based upon such input (cf. [44]). But what happens when unfolding experiences turn out to be entirely predictable? How does event segmentation happen in repeated experiences?

Outside the laboratory, events are seldom isolated experiences; instead, they often partially repeat in some form. During repeated viewings of the same movie stimulus, human region-specific cortical activity increasingly and reliably begins to precede event boundaries [45]. Indeed, repetition-enhanced activation is also seen behaviourally, with better memory performance relating to increased activation in the hippocampal formation when participants are presented with repeated stimuli (short movies, face-scene pair [46,47], respectively). Even 3-year-old children remember novel object–name pairings for longer when reading the same narrative three times versus when reading three different narratives [48].

Thus, in contrast to typical event segmentation theory, event bounding and segmentation may occur due to recognized repeating activity [49]. Here, then, we use our corridor maze to explore event segmentation while watching an agent move around a spatial environment. The corridor maze offers a periodic repetition (straight corridors and turning at the end of corridors) that allows us to examine how such environments lead to event segmentation, and how this segmentation might change over repeated maze segments and viewings. In this way, our work begins to elucidate how the formation of event units through event segmentation can occur comparably across species, further bridging the neurobiological mechanisms of memory studied in non-human animals to human-oriented event cognition theories [1].

## 2. Experiment 1

For experiment 1 we were inspired by previous work in rats (figure 1*a*). Participants watched an agent traverse a fixed path in the corridor maze from a first-person perspective. Movies and texts can often contain cuts or ‘jumps’ in spatio-temporal context which impact segmentation (e.g. [8,35]). However, such jumps in spatio-temporal context are rare in real-world situations, deviating from the typical temporal continuity of an organism’s waking phenomenology. Thus, our stimuli contained no spatio-temporal jumps and were devoid of narrative. Finally, our starting hypothesis was that people would segment more at turns based on the above-mentioned research (e.g. [27–29,37]).

### 2.1. Methods

#### 2.1.1. Participants

Eighty-six participants were recruited online from the Durham University and Newcastle University participant pool, and nearby community. They received course credits for their participation where applicable. All participants for all experiments provided informed consent, acknowledging that they had typical or corrected-to-typical eyesight. All experiments adhered to institutional guidelines and were approved by the local ethics subcommittee at Durham University (29 July 2022; reference: PSYCH−2022−01−11T12_31_41-rgrv95) and separately at Newcastle University (31 October 2022, reference: 25515/2022). An *a priori* power analysis, G*Power 3.1.9.7 [50], suggested a minimum sample size of 30 participants (two-tailed paired *t*‐test, *dz* = 0.5, α = 0.05, β = 0.75). After two separate rounds of data collection from Durham and Newcastle, respectively, each recruiting over the minimum power estimate to account for potential outliers and online testing, data was pooled as participants had experienced the same experimental procedure for the segmentation task. Although, in data collection round two, participants were asked why they pressed the spacebar key, after the segmentation task. One participant was excluded as they made no presses and failed to provide a typed response as to why they did not press, suggesting technical difficulty. Finally, two further outlier cases were excluded based on their high key press count and quartiles, where *k* = 2.07 [51], thus for experiment 1 the data from 83 participants were analysed (60 female, 18–30 years, *M*_age_ = 20.50, s.d._age_ = 3.26).

#### 2.1.2. Materials

All virtual environments were constructed in Unity (2021.3.7f1, Unity Technologies). This was done using the ‘CineMachine’ package and all videos were rendered (30 frames s^−1^, at least 960 × 540 resolution, 16 : 9 aspect ratio, MP4), displaying a maze comprising six corridors from the first-person perspective along a predetermined path for experiment 1 (figure 1*b*; electronic supplementary material, video 1; lasting 60 s). All experimental video stimuli can also be obtained on the open science framework database (https://osf.io/6swzd/?view_only=2312e2b7c6c5425ea0e9c579b3fba8b8). We used PsychoPy (v. 2021.2.3., PsychoPy^®^) to create and structure the experimental proceedings (then uploaded onto the pavlovia.org server (Pavlovia^®^) to be completed online by participants.

#### 2.1.3. Procedure

Participants were shown a start screen which stated that the video would begin next, including the task instructions (figure 1*c*): ‘When you feel one meaningful unit of activity ends and another begins, we ask you to press the “SPACEBAR” key’. They were given no prior instructions as to what may constitute a ‘meaningful unit of activity’, and were required to click the screen to begin, before subsequently being shown the video stimulus (there were no practice trials). While videos were playing, only presses of the spacebar were recorded. Participants were entirely passive throughout the duration of stimuli (i.e. they had no control over video speed and could not skip to the next screen before the stimulus had ended). For experiment 1, the same video stimulus was repeated; this was after participants were told that another video would begin next and were reminded of the task instructions, again having to click the screen before the following stimulus was shown. After the segmentation task, some participants were asked to complete a short, typed answer (approx. 60 s) in response to: ‘in your own words, could you briefly describe why you pressed the space key or why you did not press the space key’ (see §2.1.1.).

#### 2.1.4. Data analysis

The system recorded the number of spacebar key presses made and the timings of such presses. Key press analyses took a within-participant approach to the number of key presses made, whereas a binning approach considered responses across participants using key press timings. We first used coarser bins of 5 s per bin (centred at every 2.5 s, 12 in total) as this roughly corresponded to the amount of time it took for the agent to traverse the length of the corridor and around the turn, approximately 5 s respectively (electronic supplementary material, video 1). Therefore, we defined ‘bin types’, being corridor/straight bins versus turn bins. For all analyses we used SPSS (2021, IBM Corp) and MATLAB (2020, The MathWorks, Inc), and all reported statistics are two-tailed tests and post hoc tests are Bonferroni corrected to account for multiple comparisons.

### 2.2. Results

**Figure 2.**
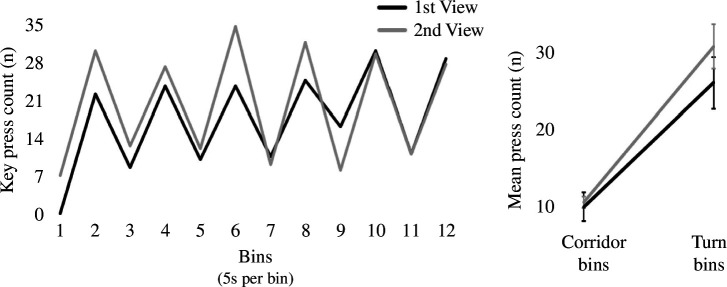
Experiment 1 event segmentation results. Left: keypress count in the corridor maze by bins. Odd bins correspond to corridors and even bins correspond to turns. Right: mean press count of corridor bins versus turn bins by viewing. Error bars denote ± 1 s.e.m. Press count for the first viewing is shown in black and for the second viewing is shown in grey.

The key presses made within the 60 s video were pooled into twelve 5 s bins. These bins alternated between approximately 5 s of moving along a corridor and approximately 5 seconds of turning a corner. A repeated measures ANOVA showed that there was no significant difference in overall key presses made in the first viewing versus the second viewing (*F*_(1,11)_ = 3.04, *p* = 0.11, *η*_p_^2^ = 0.22). There was also no overall interaction between viewing and presses made in corridors versus turns (*F*(1,11) = 4.29, *p* = 0.063, *η*_p_^2^ = 0.28). However, post hoc tests showed that within the first viewing, significantly more presses were made in turns (*M* = 25.83) relative to corridors (*M* = 9.83, *p* < 0.001; figure 2). And similarly, within the second viewing, significantly more presses were also made in turns (*M* = 30.50) relative to corridors (*M* = 10.42; *p* < 0.001). Finally, while key presses in corridors were comparable across viewings (*p* = 0.76), pressing significantly increased from the first viewing to the second viewing in turns (*p* = 0.022).

### 2.3. Discussion

In accordance with our hypothesis, the results suggested evidence of more segmentation occurring in turns relative to corridors. There was also evidence to suggest that segmentation behaviour changed across viewings. Thus, in consideration of these results, along with the worded responses of why participants segmented (echoing the behaviour; electronic supplementary material, table S1), and the implicit evidence from spatial cognition studies, e.g. [27–29,37], it provides good support that turns around spatial boundaries can cue segmentation.

## 3. Experiment 2

Previous segmentation studies have shown that detection of action-related change of actors/agents is sufficient to drive segmentation (e.g. [52,53]). We next questioned how essential the inner spatial boundaries were in influencing segmentation. To this end, we used an open-field maze (electronic supplementary material, video 2; figure 1*d*), where the agent traversed the same fixed path as in the corridor maze but there were no inner boundaries present. In other words, the open-field maze controlled for the action-related change of the agent to elucidate what role the periodic inner boundaries play in event segmentation cognition. Additionally, in a separate maze, we had the agent traverse in a continuous manner along an elongated corridor (electronic supplementary material, video 3; figure 1*e*). This controlled for the visual cues of a corridor straight to elucidate what role the action change related to the agent’s turn had on event segmentation cognition.

### 3.1. Methods

#### 3.1.1. Participants

Forty-four participants were recruited for experiment 2 across the Durham University participant pool and nearby community. For the open-field maze analyses two outliers were excluded based on their high key press count and quartiles, where *k* = 2.07 [51], resulting in the data from 42 participants (32 female, 18–32 years, *M*_age_ = 20.81, s.d._age_ = 3.10) being considered for the analyses. This included analyses made between the corridor maze, open-field maze and the elongated corridor. For analyses within the elongated corridor, a further five participants were excluded based upon the same criteria, resulting in the data of 37 participants being analysed (27 female, 18–32 years, *M*_age_ = 20.89, s.d._age_ = 3.27).

#### 3.1.2. Materials

As mentioned earlier, virtual mazes were constructed in Unity (see §2.1.2.). The open-field maze was the same dimensions as the corridor maze; however, no inner boundaries were present, and the agent traversed the same path as in the corridor maze (electronic supplementary material, video 2; lasting 60 s). The elongated corridor was simply one elongated corridor arm of the corridor maze, where the agent continuously traversed until the end boundary (electronic supplementary material, video 3; lasting 60 s).

#### 3.1.3. Procedure

The experimental procedure similarly followed that of experiment 1 (see §2.1.3.). However, after the second stimulus viewing, participants completed the segmentation task in relation to the other maze, again viewing that stimulus twice. Thus, for experiment 2 participants viewed a total of 4 videos with breaks in between them. The order in which the maze was experienced first was equally counterbalanced across participants.

#### 3.1.4. Data analysis

When analysing the open-field maze separately, we implemented the approach used in experiment 1 (§2.1.4.). There was a significant strong correlation between the segmentation of those participants who experienced the open-field maze first and those that experienced the elongated corridor first (press count of 5 s bins; first viewing: *r*_(10)_ = 0.78, *p* = 0.003; second viewing: *r*_(10)_ = 0.81, *p* = 0.001). This suggested that the maze order viewing in experiment 2 had a minimal effect upon segmentation behaviour for the open-field maze. In the comparison between the corridor maze and open-field maze stimuli, we wanted to understand whether presses were distributed equally around turns. In this way, we implemented finer-grained binning of 1 s per bin (centred at every second) and focused specifically upon bins around the turn. There was a total of 9 bins per turn, ranging from −4 to 4, where negative values denoted the agent transitioning from the preceding corridor into the turn, and positive values denoted the agent transitioning from the turn into the subsequent corridor. Due to the increased resolution of the binning, these analyses considered data across all turns and viewings. A data-driven follow-up analysis considered whether pressing in the two peaks bins around the turn, i.e. those bins with the greatest mean pressing count (essentially, turn ‘start’ and ‘end’ bins; corridor maze: bin −3 and 1; open-field maze: −3 and 3) was largely driving segmentation overall at turns. Thus, the mean of start-end bins were compared with the mean of those bins in between them, i.e. turn ‘middle’ bins (corridor maze: bins −2 to 0; open-field maze: bins −2 to 2).

To assess whether and how segmentation changed over time across the corridor maze and the open-field maze data, we divided these mazes into thirds. A given third was constituted by press counts from a left-turn bin and right-turn bin and their preceding corridor/straights (using coarser-grained binning). Thus, this allowed examination of how segmentation behaviour evolved within viewings (within groups), across viewings (within groups) and between groups. The maze order experience in experiment 2 had no effect on press counts across thirds and viewing (*F*_(2,12)_ = 0.84, *p* = 0.46, *η*_p_^2^ = 0.12, all *p* ≥ 0.16). Finally, we asked where changes in segmentation were reliably occurring across viewings, beyond turn bins *per se*. Within participants, their key press timings were coded as to whether the timings corresponded to one of the following categories: corridor/straight (corridor maze: bins −4 and bin 3 onwards; open-field maze: bins −4 and 4 onwards), turn middle/end of maze (corridor maze: bins −2 to 0; open-field maze: bins −2 to 2; and last 5.5 s of each stimuli), turn onset (corridor maze and open-field maze: bin −3), turn offset (corridor maze: bins 1 and 2; open-field maze; bins 2 and 3) and lastly, both turn onset and offset of the same turn. Once coded, the proportion of each category’s contribution to the total number of presses made was calculated, within subject. Proportion change was the subtraction of the first viewing proportion from the second viewing proportion completed for each category.

### 3.2. Results

#### 3.2.1. Segmentation in the open-field maze

**Figure 3.**
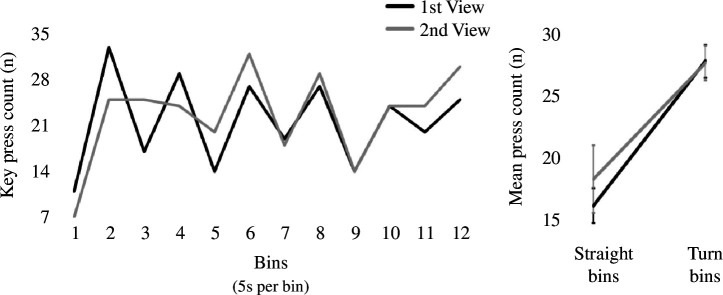
Experiment 2 open-field maze event segmentation results. Left: key press count in the open-field maze by bins. Odd bins correspond to straight paths made by the agent and even bins correspond to turns made by the agent. Right: mean press count of straight path bins versus. turn bins by viewing. Error bars denote ± 1 s.e.m. Press count for the first viewing is shown in black and for the second viewing is shown in grey.

As for experiment 1 key presses made within the 60 s video were pooled into twelve 5 s bins. A repeated measures ANOVA showed that there was no significant difference in overall key presses made in the first viewing versus second viewing (*F*_(1,5)_ = 0.36, *p* = 0.57, *η*_p_^2^ = 0.07). There was also no overall interaction between viewing and pressing made in straights versus turns (*F*_(1,5)_ = 1.00, *p* = 0.36, *η*_p_^2^ = 0.17). However, post hoc tests showed that more presses were made in turns of both the first and second viewings (figure 3; *M* = 27.50, *M* = 27.33; respectively) versus in straights (*M* = 15.83, *M* = 18.00, *p* = 0.004, *p* = 0.02; respectively). Moreover, there was no difference in pressing in turns and straights across viewings (*p* = 0.94, *p* = 0.30; respectively). Thus, similarly to experiment 1 using the corridor maze, more segmentations were made when the agent made turns relative its straight path in the open-field maze.

#### 3.2.2. Comparison of segmentation in the elongated corridor versus the corridor and open-field mazes

Unlike the corridor and open-field mazes the agent made no turns in the elongated corridor, so we used key press timings (§2.1.4.) to make comparisons between the stimuli. There was an overall effect regarding the key presses data in experiment 2 across mazes and viewings (χ^2^_(3)_ = 41.22, *p* < 0.001). Post hoc analyses showed that in the open-field maze there was no difference in pressing from the first viewing (*M* = 6.19, Md = 6.00, s.d. = 4.39) to the second viewing (*M* = 6.48, Md = 6.00, s.d. = 3.92; *Z* = −1.14, *p* = 1.00). Similarly, within the elongated corridor there were no differences across viewings (first viewing: *M* = 2.38, Md = 1.00, s.d. = 5.33; second viewing: *M* = 3.48, Md = 2.00, s.d. = 6.00; *Z* = −0.38, *p* = 1.00; electronic supplementary material S1). However, there were significantly more presses made in both the first and second viewings of the open-field maze compared with that of the elongated corridor (*Z* = 4.65, *p* < 0.001, *Z* = 3.89, *p* = 0.001; respectively). Moreover, there were significantly more presses made in the corridor maze (*M* = 5.54, Md = 5.50, s.d. = 3.85) versus the elongated corridor averaged across viewings (*M* = 2.93, s.d. = 4.43, Md = 1.50; *Z* = −4.47, *p* < 0.001). Thus, in summary, more segmentations were made in the corridor and open-field mazes where the agent made turns relative to the elongated corridor.

#### 3.2.3. Comparison of segmentation in the corridor maze versus the open-field maze

**Figure 4.**
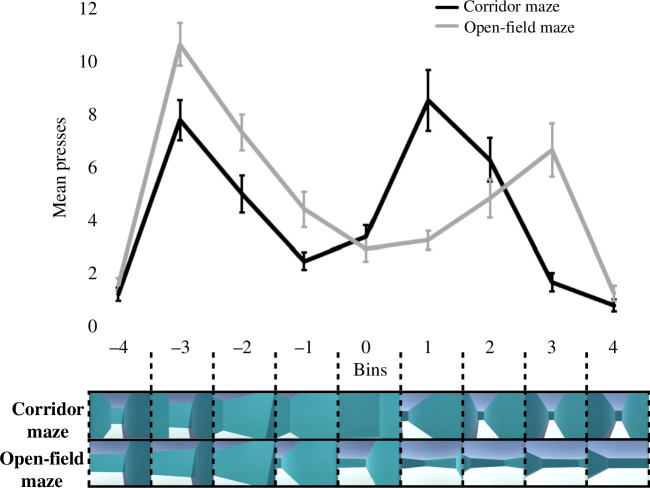
Event segmentation in the corridor maze versus the open-field maze. Upper: mean press count by 1 s time bins focused upon turns. Error bars denote ± 1 s.e.m. The corridor maze and open-field maze are displayed in black and grey, respectively. Lower: displays an example left turn sequence of frames by bin for both the corridor maze and the open-field maze.

As more segmentation was made in turns relative to corridors (straight paths for the open-field maze), we next asked whether segmentation was equally distributed around turns. To address this we focused on nine bins of 1 s per bin around turns (see §3.1.4.). A mixed repeated measures ANOVA showed that there was no overall significant difference between the corridor maze group and the open-field maze group (*F*_(1,34)_ = 2.14, *p* = 0.15 *η*_p_^2^ = 0.06). However, there was a significant interaction between bin and group (*F*_(3.78, 128.60)_ = 9.33, *p* < 0.001, *η*_p_^2^ = 0.22). Post hoc analyses showed that there was a significant increase in mean pressing as the agent transitioned from the corridor (bin −4; *M* = 1.21) into the turn (bin −3; *M* = 7.79, *p* < 0.001). And similarly in the open-field maze, there was a significant increase in mean pressing as the agent transitioned from a straight path (bin −4; *M* = 1.58) into a turning action (bin −3; *M* = 10.67, *p* < 0.001; figure 4).

In regard to the agent ending the turning action, a significant decrease in mean pressing was seen in the corridor maze when the agent was exiting the turn (bins 1 and 2; *M* = 8.54 and 6.29, respectively) into the following corridor (bin 3; *M* = 1.67; bin 1 versus bin 3, *p* < 0.001; bin 2 versus bin 3, *p* < 0.001; bin 1 versus 2, *p* = 0.068). Whereas in the open-field maze, this significant decrease in mean pressing occurred later. Where the agent finished its turning at bin 3 (*M* = 6.67) and begun its straight path at bin 4 (*M* = 1.25, *p *< 0.001, see electronic supplementary material, table S2 for further within-group comparisons and electronic supplementary material, table S3 for within-bin comparisons). This suggested that there were discrete moments in which elevated segmentation was occurring in both mazes.

We next asked whether pressing at the agent’s turn onset and turn offset, start and end bins, respectively, were driving segmentation behaviour overall at turns relative to bins in between them (i.e. turn middle bins; see §3.1.4.). A mixed repeated measures ANOVA showed no significant difference across the corridor maze and open-field groups (*F*_(1,34)_ = 1.13, *p* = 0.30, *η*_p_^2^ = 0.03). There was also no overall interaction between group and turn start-end bins versus turn middle bins (*F*_(1,34)_ = 0.10, *p* = 0.75, *η*_p_^2^ = 0.003). However, post hoc tests showed that for the corridor maze there were significantly more presses made in turn start-end bins (*M* = 8.17) relative to turn middle bins (*M* = 3.61, *p* < 0.001). Similarly for the open-field maze, significantly more presses were made in turn start-end bins (*M* = 8.67) relative to turn middle bins (*M* = 4.55; *p* < 0.001). Yet, there was no difference in pressing across the maze groups in turn start-end bins and turn middle bins (*p* = 0.70, *p* = 0.063; respectively).

To summarize, segmentation was overall similar between the corridor maze and the open-field maze, with discrete moments of elevated segmentation occurring in both mazes. In essence, this demarcated the start and end of turn and corridor (straight path) events, with pressing in these start and end bins largely driving segmentation behaviour overall at turns.

#### 3.2.4. Comparison of segmentation over time between the corridor maze and the open-field maze

In experiment 1 the results suggested that segmentation behaviour changed across viewings in the corridor maze. Despite the key difference between the corridor maze and open-field maze being the presence of inner boundaries, both stimuli had periodic structure to them, where turning was the important action in both mazes. Therefore, we next asked whether and how behaviour changed across groups within and across viewings (see §3.1.4.). A mixed repeated measures ANOVA showed no overall significant differences between viewing, nor thirds, nor group (all *F* ≤ 3.75, *p* ≥ 0.07). There were also no significant overall interactions between thirds and group, nor viewing and group (all *F* ≤ 1.18, *p* ≥ 0.33). However, there was an overall significant three-way interaction between thirds, viewing and group (*F*_(2,20)_ = 3.65, *p* = 0.045, *η*_p_^2^ = 0.27; electronic supplementary material S2.). Post hoc tests showed no differences between groups (all *p* ≥ 0.30), and also no differences in the open-field maze across maze thirds within and across viewings (all *p* ≥ 0.27).

In the corridor maze group, post hoc tests showed that there were no differences across within maze thirds in the second viewing (all *p* ≥ 0.60). Within the first viewing, there was also no difference between the first third of the maze (*M* = 14.00) versus the middle third (*M* = 17.63, *p* = 0.18). However, within the first viewing, mean pressing significantly increased from the first third of the maze to the final third (*M* = 21.88, *p* = 0.023), and from the middle third of the maze to the final third (*p* = 0.027). Moreover, mean pressing was significantly greater in the first maze third of the second viewing (*M* = 19.63) relative to the first viewing (*p* = 0.005). And similarly, mean pressing was also significantly greater in the middle maze third of the second viewing (*M* = 22.25) relative to the first viewing (*p* = 0.03). There was no difference in the final maze of the second viewing (*M* = 19.50) relative to the first viewing (*p* = 0.30).

We finally asked where possible changes were occurring across viewings by leveraging the findings showing discrete moments of elevated segmentation (§3.1.4), In the open-field maze group (*n* = 42), we found no proportion changes from the first viewing to the second viewing in any discrete moments relative to chance (all *p* ≥ 0.094, one-sample Wilcoxon signed rank test, chance being zero). However, in the corridor maze group (*n* = 83), we found that only the proportion of pressing in both the turn onset and turn offset of the same turn increased from the first viewing to the second viewing, significantly differing from chance being zero (*M* = 4.9%, Md = 0%, s.d. = 15.9%; *Z* = 2.62, *p* = 0.009, *r* = 0.29; all other discrete moments *p* ≥ 0.083). These results were upheld when repeating the analyses and limiting to only those participants who at least had made the same number of presses across viewings if not more (open-field maze group: *n* = 28, all *p* ≥ 0.091; corridor maze group: *n* = 54, *M* = 6.1%, Md = 0%, s.d. = 15.1%; *Z* = 2.84, *p* = 0.004, *r* = 0.39, all other discrete moments, *p* ≥ 0.061).

## 4. General discussion

Accumulating evidence demonstrates that episodic memory and spatio-temporal related perceptual estimations depend upon event segmentation (e.g. [10,25,33]). This makes it an important neurocognitive process to understand. Previous research suggested that movement of agents/actors can cue segmentation [52,53]. Generally, this is better understood as action-based event segmentation where one is especially perceptive of goal-oriented intentions [4,52,53]. Indeed, segmentation is largely similar when the same actions (e.g. doing the laundry) are shown from a first-person or third-person perspective [54]. In other words, despite these stimuli vastly differing in the quantity, availability and fluctuation of sensory information, there is overall similar event segmentation, suggesting that it can be viewpoint invariant and can occur on a more conceptual level [54].

Less segmentation in the elongated corridor is unsurprising according to action-based segmentation theory, because there was no action-based change by the agent. Conversely, this also explains why more segmentation was made at turns versus corridor/straights in the corridor maze and the open-field maze, being due to the agent’s action-based change. Moreover, segmentation in these turn onset and offset peak points, compared with moments in between them, were probably emphasized because perceptual changes in the agent’s movement and optic flow accompanied the action-based change in both mazes (figure 4). Even in animations using two-dimensional shapes, more bursts of motion-related changes led to enhanced segmentation, associated with coarser-grained event segmentation [53]. As the agent performed the same actions in the corridor maze and open-field maze, the observation of similar segmentation occurring argues that, despite some differences in lower-level visual information [54], the open-field maze was a good control to elucidate what roles the spatial boundaries played in segmentation, beyond action-based segmentation explanations.

A robust difference between the corridor maze and open-field maze was that the turn offset peak in segmentation lagged by 2 s. The lack of boundaries probably resulted in greater ambiguity of when the agent’s turning action ends, peaking once forwards movement towards the outer boundary was detected. In the corridor maze, however, concavity of the spatial boundaries may constrain segmentation behaviour (cf. [55]), making it feel like the agent’s turn was ending and a path in a new separate corridor was beginning (figure 4). Thus, many participants did not have to wait to detect onwards movement by the agent, with the presence of boundaries possibly allowing corridor maze participants to better anticipate the impending turn offset event boundary.

Further differences between the corridor maze and open-field maze became apparent when examining behaviour over time. Increasing segmentation within and across viewings in the corridor maze is not well accounted for by event segmentation theory [7], as strong views of this theory should predict that segmentation would decrease over time, if no prediction-errors are experienced. Yet, due to a combination of the above-described action-based explanations and periodic nature of the mazes, it is understandable that consistent segmentation behaviour could occur over time. Such consistency was observed in the open-field maze within and across viewings, and indeed given such simplistic stimuli, one concern may be that demand characteristics was determining behaviour. Importantly, however, only in the corridor maze did increased segmentation occur within and across viewings, with an increased proportion of segmentation responses being made specifically at both turn onsets and offsets belonging to the same turn (from the first to the second viewing). This argues against simpler explanations of behaviour (e.g. action-based segmentation or demand characteristics), instead, suggesting that event bounding can be driven by learning of recognized repeating activity [49].

Avrahami and Kareev [49] suggested a cut hypothesis for event formation. They showed that when people experienced short sequences of stimuli (embedded within longer sequences) that repeated in several different sequence contexts, the short sequences began to be cognized events with clear beginnings and ends. Our results are not in complete agreement with the cut hypothesis argued in this way. As the changes in segmentation that we observed over time in the corridor maze occurred in very similar spatio-temporal contexts, we posit instead that, in some situations, recognized repeating activity unfolding in similar contexts can become more ‘meaningful’ to an individual over time (and or with more experience), facilitating the formation of event units for memory. Importantly, this view is conceptually consistent with the well-established function of pattern separation and its role in recognition memory [56]. Pattern separation, associated with the dentate gyrus of the hippocampus, is a process where similar overlapping inputs are discretized into non-overlapping outputs [56]. Therefore, an intuitive hypothesis is that during repetitive experiences hippocampal-dependent pattern separation computations may be especially facilitated at event boundaries (cf. [45,57]). Forming experimental designs that combine segmentation task behaviour [6,14,45], along with tasks that have teased apart pattern-separated items from contextual source during memory retrieval [58], would be one possible way to begin testing such an idea. Thus, event segmentation theory works particularly well during experiences that are novel and or surprising [7]. Whereas segmenting events via recognized repeating activity can function during very similar experiences (potentially facilitating pattern separation), and together they can operate in a complementary manner to form successful episodic memories in animals. However, this raises the question of what is it about the periodic spatial boundaries of the corridor maze that led to these changes in segmentation over time?

From the agent’s first turn in the open-field maze one can ascertain the entirety of the spatial layout. Not only that, but the distal walls important for setting allocentric reference directions and navigation [59,60] can act as constant cues to keep track of where the agent is in the maze, resulting in ongoing dissimilar input. In contrast, distal wall cues could not be used in the same way in the corridor maze, as the inner boundaries occluded the viewing of other corridors, probably affording each corridor to feel like a separate scene [55] but with the input being very similar in nature. One, then, requires more turn experiences to ascertain the entirety of the spatial layout, leading to the realization that the spatial context (and agent) was repeating itself. Some participant explanations as to why they segmented, were consistent with this view: ‘Because the circuit began to repeat’ [participant 4820]. ‘The loop started again from the beginning (the loop being turning left then right)’ [participant 4664]. To summarize, increasing segmentation over time in the corridor maze occurred because unlike in the open-field maze (i) participants could not ascertain the entirety of the spatial layout from the first turn onwards. (ii) Inner boundaries prevented distal wall cues from effectively being used to keep track of where the agent is in the maze. (iii) More experience is then required for participants to realize that the agent and the spatial context repeats itself. Finally, as input (of corridors/turns) was also very similar in the corridor maze this may have reflected a greater demand for pattern separation (hence increasing segmentation) compared with the open-field maze.

To come full circle, grid-cells were seen to form discrete spatial representations for each corridor in the corridor maze [37] (figure 1). Indeed, converging evidence from rodents and humans show that spatial boundaries influence the activity of cells in the hippocampal formation, facilitating spatial representations [61–64]. Therefore, we acknowledge that direct cross-species comparisons are limited from these experiments alone. Yet, we do argue that the findings here provide an important step towards better bridging the event segmentation literature in humans to the rapidly developing non-human animal research in systems and behavioural neuroscience. Specifically, we elucidate that turns can cue segmentation and elucidate when such segmentations may occur (especially around spatial boundaries). Consequently, this is informative as it provides a clearer framework for the formation of event units across species. An example area of research that benefits from this is hippocampal-dependent replay, the reactivation of previously activated neural sequences, occurring in both rodents [65–67] and humans [68]. While many rodent studies have observed replayed trajectories of short traversed paths on linear tracks (e.g. [65,66]), it is speculative to suggest that in all such cases the activity is reflective of an event unit. Recently, however, flexible replay around one or more barriers within the same physical location was shown in rats [69], which in light of this work would argue that the content of the replay is more representative of previously experienced event units. Future work may also begin to utilize implicit methods that allow behavioural assessment of learning and memory in rodents (e.g. [70,71]), and further explore how turn-based segmentation around spatial boundaries may influence episodic-like memory in these animals.

In conclusion, our experiments demonstrate that turns during navigation can cue segmentation of events. The simplicity of the experimental design, when controlling for action-based change, also provided insight into how periodic spatial boundaries contributed to a lesser-developed theory of event segmentation. Specifically, that segmentation can increase over time, probably becoming more meaningful due to recognized repeating activity. This potentially unites functions of event segmentation and pattern separation to ultimately advance our understanding of episodic-like memory in non-human animals and episodic memory in humans.

## Data Availability

The data that support the findings of this manuscript are available on the Open Science Framework repository [72]. Supplementary material is available online [73].
